# Spontaneous splenic rupture: a sporadic presentation of tuberculosis—a case report

**DOI:** 10.1093/jscr/rjaf1103

**Published:** 2026-01-20

**Authors:** Zeru A Sambi, Mehari A Tsegay

**Affiliations:** Department of Surgery, Hepatopancreatobiliary Surgery Unit, Hayat Hospital Medical College, Bole Subcity, Mecon, 1534, Addis Ababa, Ethiopia; Department of Surgery, Ayder Comprehensive & Specialized Hospital, Mekelle University, Mekelle Romanat Square, 1871, Mekelle, Ethiopia

**Keywords:** spleen, tuberculosis, spontaneous rupture, HIV, splenectomy

## Abstract

Atraumatic or spontaneous splenic rupture is a rare but life-threatening emergency. Common causes include hematologic malignancies and infections, but tuberculous involvement of the spleen is exceedingly uncommon. To our knowledge, this represents the third reported case of spontaneous rupture of a tuberculous spleen. We report a 50-year-old human immunodeficiency virus (HIV) -positive woman who presented with cough, fever, and abdominal distension, with abdominal examination suggesting peritoneal irritation and laboratory tests showing severe anemia. Emergency laparotomy revealed a ruptured spleen with 500 ml of hemolyzed blood, and splenectomy was performed. Histopathology confirmed splenic tuberculosis. The postoperative course was uneventful. Splenic tuberculosis typically occurs in disseminated disease among immunocompromised individuals. Fever of unknown origin is the commonest presentation; spontaneous rupture is exceptionally rare and difficult to diagnose. Although antitubercular therapy is the mainstay of treatment, hemodynamically unstable patients require surgery. Spontaneous splenic rupture should be considered in immunocompromised patients with an acute abdomen, as early recognition is lifesaving.

## Introduction

Spontaneous splenic rupture (SSR)—defined as splenic rupture occurring in the absence of trauma—is a rare but life-threatening abdominal emergency. Tuberculosis (TB) continues to be a major global health challenge, with millions of new infections each year and a disproportionately high burden in low-income countries. Extrapulmonary TB accounts for up to one-third of all cases and is particularly common among immunocompromised patients, including those with HIV infection [1].

Splenic involvement in TB is uncommon and most often occurs as part of disseminated or miliary disease. Its clinical presentation is frequently vague, with fever of unknown origin being the most typical manifestation. Other features may include constitutional symptoms, splenomegaly, portal hypertension, and, rarely, splenic rupture [2]. Because of its nonspecific presentation, diagnosis relies on a combination of hematologic tests and imaging modalities such as ultrasonography, contrast-enhanced CT, or MRI, which may reveal characteristic splenic lesions [3]. Definitive confirmation requires histopathological or microbiological evidence.

Antitubercular therapy (ATT) remains the mainstay of management for splenic TB. However, when splenic rupture leads to ongoing hemodynamic instability, urgent surgical intervention becomes essential [3, 4].

Spontaneous rupture of a tuberculous spleen is extremely uncommon, with only two previous cases reported [3]. Here, we present a rare case of SSR secondary to splenic TB in an HIV-positive woman, highlighting the diagnostic challenges and management considerations in this unusual presentation.

This case is reported in accordance with the SCARE 2020 guidelines [5].

## Case presentation

A 50-year-old HIV-positive woman presented with a 5-day history of progressive abdominal distension and fatigue, and a 2-week history of cough and shortness of breath. She denied any trauma.

On examination, she was cachectic, pale, tachypneic, and tachycardic, but normotensive. Chest examination revealed right-sided crepitus. Her abdomen was distended with generalized tenderness and guarding suggestive of peritoneal irritation.

### Investigations

**Figure 1:**
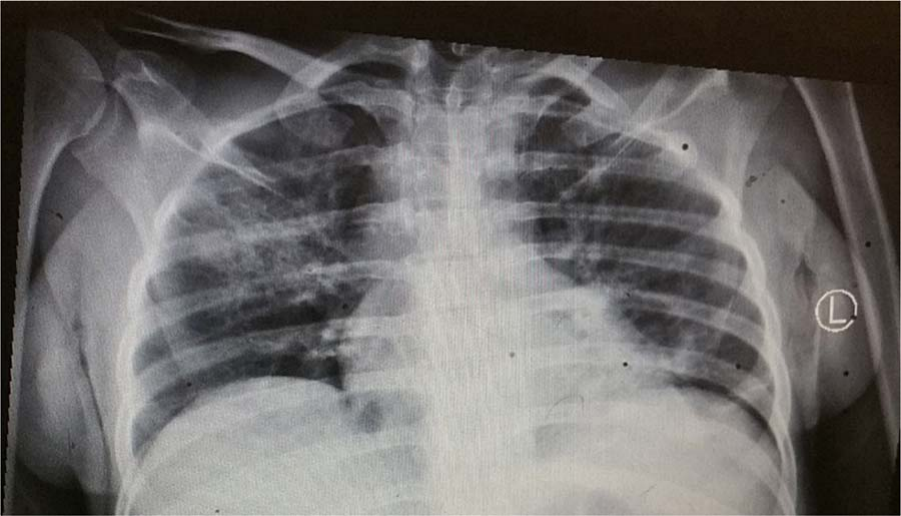
Chest X-ray showing consolidations in the right lung.

The patient had a normal white cell count of 4600/mm^3^, but the hemoglobin level was low at 5.8 g/dl. The serum electrolytes, renal function, and liver function tests were all within the normal range. Chest X-ray showed right lung consolidation (Fig. 1). No free air was seen under the diaphragm.

### Management and outcomes

**Figure 2:**
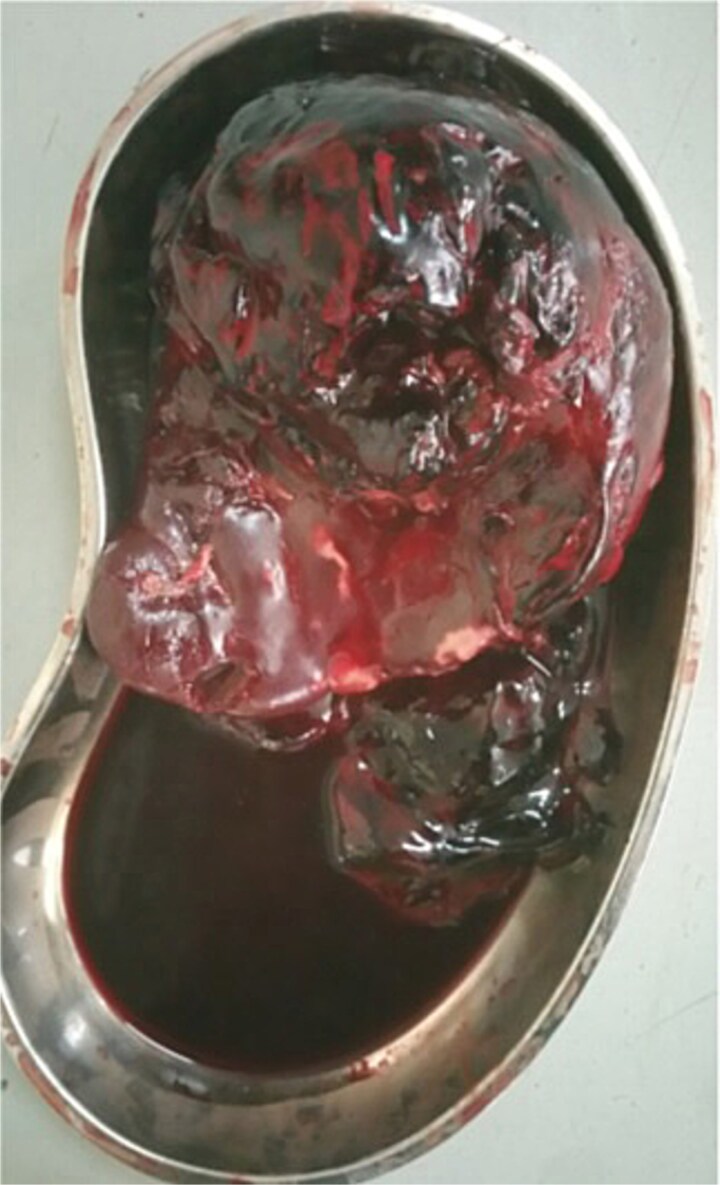
Intraoperative picture showing a ruptured spleen.

Given her peritoneal signs, she underwent emergency exploratory laparotomy via a midline incision. Approximately 500 ml of hemolyzed blood was found in the peritoneal cavity. The spleen was ruptured at the lower pole with multiple lacerations and a large subcapsular hematoma (Fig. 2). Given the patient’s immunocompromised status, severe anemia, and splenic lacerations involving the entire parenchyma (Fig. 2), we proceeded with splenectomy as the definitive and safest management option.

**Figure 3:**
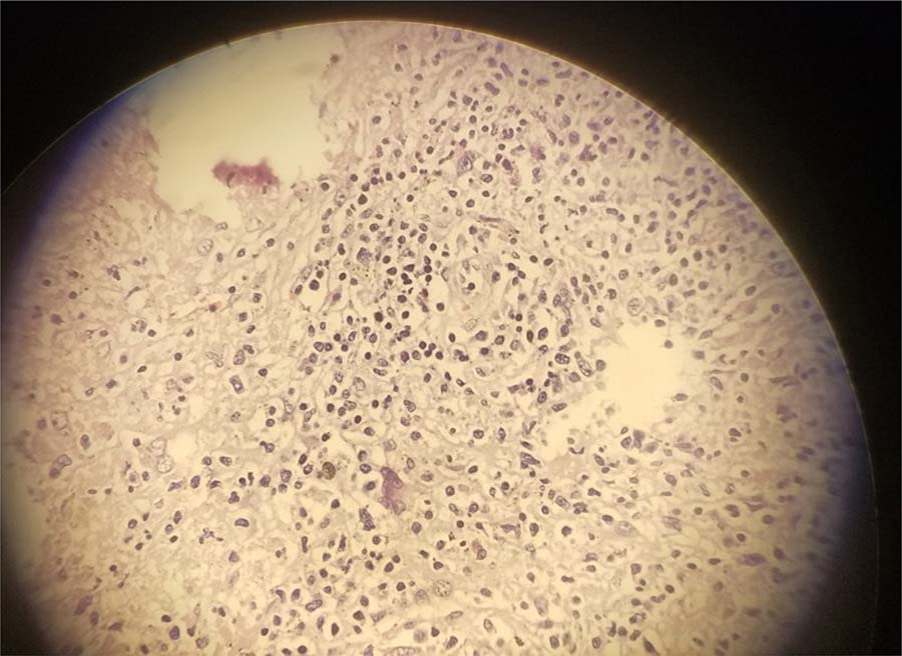
Microscopic slide of the specimen, which is consistent with splenic TB.

Histopathology revealed multiple epithelioid granulomas with central caseation and Langhans giant cells, confirming splenic TB (Fig. 3). Postoperatively, she recovered well and was discharged in good condition. She was started on ATT (isoniazid, rifampicin, pyrazinamide, and ethambutol for 2 months, followed by isoniazid and rifampicin for 4 months). She remained stable on follow-up.

## Discussion

### Epidemiology

TB is a major global health priority, causing an estimated 10.8 million new cases and 1.25 million deaths in 2023. It remains the leading cause of death from a single infectious agent worldwide [1]. The burden of disease falls disproportionately on low- and middle-income countries, including Ethiopia, which ranks among the 30 high-TB-burden nations with an estimated incidence of 146 per 100 000 population [3].

### S‌SR and splenic TB

SSR is a rare clinical entity, defined as rupture in the absence of significant trauma, with an estimated incidence of 0.1%–0.5% [5]. It is categorized as either “true” (occurring in a histologically normal spleen) or “pathologic” (occurring in a diseased spleen). The etiologies of pathologic SSR are diverse, including infectious, neoplastic, and hematologic malignancies. Infectious causes such as malaria and infectious mononucleosis are well-documented; however, splenic TB as a cause for SSR is exceptionally uncommon [6].

### Pathophysiology

The pathophysiology of SSR typically initiates with the development of a subcapsular hematoma. It is postulated that minor mechanical stress from routine physiological activities may precipitate splenic hemorrhage, infarction, congestion, and focal necrosis, culminating in capsular rupture. Splenic rupture leads to the opening up of splenic sinusoids, which in turn leads to torrential hemorrhage, as the sinusoids lack the musculature for vasospasm and control of the bleeding [7].

### Clinical presentation

Pulmonary TB is the most common form of the disease and is transmitted via airborne droplet nuclei, particularly during coughing [8]. Extrapulmonary TB accounts for 15%–20% of all cases, with abdominal TB comprising 3%–11% of these cases. Abdominal TB manifests in diverse forms, including lymphadenitis, gastrointestinal, peritoneal, and visceral involvement, with concurrent pulmonary disease in ~15% of cases. Splenic TB arises as a component of disseminated (miliary) TB or, less frequently, in isolation. In miliary TB, the spleen ranks as the third most commonly involved organ after the lungs and liver [3]. The majority of splenic TB cases occur in profoundly immunocompromised hosts, as in our patient.

Splenic TB is more common in men aged 19–53 years. Risk factors include immunosuppression, prior pyogenic splenic infections, and a history of splenic trauma. Primary (isolated) splenic TB remains particularly challenging to diagnose due to its nonspecific clinical features, including fever (82.3%), fatigue and weight loss (44.1%), and splenomegaly (13.2%–100%) [2]. Additional manifestations include splenic rupture, portal hypertension, and a fulminant variant characterized by rapid progression of fever, cachexia, hemorrhage, and sepsis [4]. In contrast, our patient—a female—presented with abdominal distention and symptoms suggestive of lower respiratory tract infection, rendering the diagnosis of SSR elusive.

### Diagnosis

Accurate diagnosis relies on a combination of hematological, radiological, and microbiological investigations. Typical findings include leukocytosis and elevated ESR; cytopenia from hypersplenism may occur. Imaging— In cases of isolated splenic involvement, chest radiography is often normal [9]. However, pulmonary involvement or mediastinal lymphadenopathy, causing mediastinal widening, may be evident. Ultrasound and contrast-enhanced CT reveal hypoechoic or hypodense splenic nodules representing tuberculomas or abscesses. Magnetic resonance imaging (MRI) may reveal peripheral enhancement with central necrosis [3].

A definitive diagnosis, however, requires histopathological and microbiological confirmation. Preoperative aspiration or core biopsy under USG or CT guidance can provide a diagnosis, potentially obviating the need for more invasive surgery [9]. Image-guided fine-needle aspiration or biopsy has about 88% sensitivity. Histology typically shows caseating granulomas with epithelioid cells and Langhans giant cells, while PCR or AFB staining confirms TB.

### Management

The cornerstone of management for splenic TB is a standard ATT regimen, which consists of a four-drug combination (isoniazid, pyrazinamide, rifampicin, and ethambutol) for the initial 2 months (intensive phase), followed by a two-drug regimen of rifampicin and isoniazid for the subsequent four months (continuation phase) [4].

In cases complicated by splenic abscess formation, surgical intervention may be required. Hemodynamically stable patients with splenic rupture can often be managed conservatively, with close monitoring and serial ultrasonography or computed tomography (CT) imaging to assess healing, which typically occurs within 2–3 weeks [10]. However, patients presenting with persistent hemodynamic instability or refractory shock despite adequate resuscitation should undergo splenectomy [3].

In this case, our patient presented with an acute abdomen, signs of peritonism and severe anemia due to splenic rupture, necessitating emergency splenectomy. Following surgery and appropriate ATT, she made a full recovery.

## Conclusion

Spontaneous rupture of a tuberculous spleen is an extremely rare but fatal complication of splenic TB. Only two prior cases have been described. It should be considered in immunocompromised patients presenting with an acute abdomen, even in the absence of a history of trauma. Awareness of this rare presentation is important, particularly in regions endemic for TB. Early recognition and prompt surgical intervention, combined with ATT, are essential for survival and favorable outcomes.

## Supplementary Material

Highlight_SSR_rjaf1103
