# Supplementary material for: Spontaneous splenic rupture: a sporadic presentation of tuberculosis—a case report
Source: J Surg Case Rep. 2026 Jan 20;2026(1):rjaf1103. doi: 10.1093/jscr/rjaf1103 (PMC12818014; doi:10.1093/jscr/rjaf1103)
Supplement: Highlight_SSR_rjaf1103 [file highlight_ssr_rjaf1103.docx]

- Splenic injury is usually associated with trauma and its rupture without trauma is referred to as spontaneous splenic rupture (SSR).
- SSR is an abdominal catastrophe characterized by hemodynamic instability which requires immediate intervention.
- The most common causes of SSR are anticoagulant use and hematologic malignancies, with tuberculosis being a rare cause.
- Splenic tuberculosis is a rare form of extrapulmonary tuberculosis that usually affects immunocompromised patients with significant levels of immunosuppression.
- Antitubercular medications have a remarkable efficacy for mitigating the symptoms but in cases of persistent shock that is unresponsive to blood product resuscitation, splenectomy should be considered.
- Although tuberculosis-induced SSR is rare, it should be considered in areas with high tuberculosis prevalence and among immunocompromised patients with hemodynamic instability.
